# Regulation of PCSK9 During Oxidized LDL-Induced Foam Cell Formation in RAW264.7 Cells

**DOI:** 10.3390/biology15151307

**Published:** 2026-08-05

**Authors:** Md Sariful Islam Howlader, Manjusri Das, Surajit Hansda, Md Afjalus Siraj, Hiranmoy Das

**Affiliations:** 1 Department of Pharmaceutical Sciences, Jerry H. Hodge School of Pharmacy, Texas Tech University Health Sciences Center, Amarillo, TX 79106-1712, USA; sarifulbd@gmail.com (M.S.I.H.); dasmanjusri7@gmail.com (M.D.); shansda@ttuhsc.edu (S.H.); 2 Department of Pharmaceutical Sciences, College of Pharmacy, Ferris State University, Big Rapids, MI 49307, USA; mdafjalussiraj@ferris.edu

**Keywords:** foam cell, Kruppel-like factor 2, proprotein convertase subtilisin/kexin type 9, GGTI298, GGPP

## Abstract

Proprotein convertase subtilisin/kexin type 9 (PCSK9) is traditionally known for regulating plasma LDL cholesterol levels via LDL receptor degradation. This exploratory study examined the association between chemically induced changes in Krüppel-like factor 2 (KLF2), a vasoprotective transcription factor, and PCSK9 expression under ox-LDL-induced conditions in RAW264.7 cells. In silico molecular docking was also performed to determine whether GGTI298 could adopt a predicted binding pose within PCSK9. KLF2 was modulated by adding GGPP (a chemical inhibitor) and GGTI298 (a chemical activator) to the monocytes. Quantitative real-time PCR and immunocytochemistry were used to assess KLF2 and PCSK9 expression. Molecular docking was performed to examine the interaction between GGTI298 and PCSK9 using computational tools. Results show that ox-LDL significantly increased PCSK9 expression in monocytes during foam cell formation. However, GGPP significantly increased PCSK9 expression. In contrast, GGTI298 markedly reduced PCSK9 expression, suggesting a negative regulatory role of KLF2. Docking studies revealed that GGTI298 binds to the PCSK9 catalytic domain with favorable binding energy, forming stable hydrogen bonds and hydrophobic interactions with key amino acid residues, indicating potential interference with PCSK9 function. GGTI298 suppresses PCSK9 expression during foam cell formation, highlighting its protective role. The dual ability of GGTI298 to enhance KLF2 expression and directly bind to PCSK9 underscores its potential as a therapeutic agent for managing foam cell formation that leads to atherosclerosis. The findings demonstrate an inverse association between KLF2 and PCSK9 expression following chemical treatment under ox-LDL-induced conditions. However, direct KLF2-dependent regulation of PCSK9, direct GGTI298–PCSK9 binding, and functional inhibition of PCSK9 were not established and require further genetic, biochemical, and functional validation.

## Simple Summary

Proprotein convertase subtilisin/kexin type 9 (PCSK9) regulates plasma LDL cholesterol levels via LDL receptor degradation. The upstream regulators of PCSK9 in monocytes remain poorly defined. We investigate the regulatory role of modulating Krüppel-like factor 2 (KLF2), a vasoprotective transcription factor, in controlling PCSK9. Foam cell formation was induced by adding ox-LDL to monocytes (RAW264.7 cells). Quantitative PCR revealed that ox-LDL significantly increased PCSK9 expression in monocytes during foam cell formation. Previous studies from our group showed that the presence of GGTI298 increased the expression of KLF2 and was associated with reduced lipid accumulation, reactive oxygen species production, and mitochondrial dysfunction during ox-LDL-induced foam cell formation of RAW264.7 cells. In the present exploratory study, we show that ox-LDL exposure increased PCSK9 expression, whereas addition of GGTI298 was associated with increased KLF2 expression and reduced PCSK9 expression. These observations suggest that altered PCSK9 expression may represent a pathway associated with the effects of GGTI298 under ox-LDL-induced conditions. However, direct regulation of PCSK9 by KLF2 and direct binding or inhibition of PCSK9 by GGTI298 require further experimental validation. The dual ability of GGTI298 to induce KLF2 expression and directly bind to PCSK9 underscores its potential as a therapeutic agent for managing foam cell formation that leads to atherosclerosis.

## 1. Introduction

The transformation of monocyte-to-foam cell is a critical cellular pivot that dictates the transition from health to pathogenesis to initiate atherosclerosis, which is no longer viewed simply as a passive accumulation of lipids within the monocytes and blocking the arterial wall; it is a progressive, chronic inflammatory odyssey. When monocytes infiltrate the subendothelial space and engulf excessive oxidized low-density lipoprotein (ox-LDL) via scavenger receptors, they transition into lipid-laden foam cells. These cells contribute to plaque progression by releasing pro-inflammatory cytokines, reactive oxygen species (ROS), and matrix-degrading enzymes that promote vascular injury and plaque instability [1,2]. Central to this metabolic disruption is the proprotein convertase subtilisin/kexin type 9 (PCSK9), an enzyme traditionally recognized for its role in hepatic lipid metabolism through the degradation of low-density lipoprotein receptors (LDLR) [3,4]. However, emerging evidence indicates that PCSK9 acts as a direct local antagonist within the vasculature. By upregulating scavenger receptors such as CD36 and LOX-1 in monocytes while simultaneously inhibiting cholesterol efflux through the downregulation of ATP-binding cassette transporter A1 (ABCA1), PCSK9 serves as a master regulator of foam cell differentiation and an amplifier of the inflammatory signaling that destabilizes atherosclerotic plaques. [2,5].

The regulation of such complex cellular transitions often falls under the control of the Krüppel-like factor (KLF) family, a group of zinc-finger transcription factors that act as molecular switches for cellular identity. KLF2, in particular, is a fundamental architect of cell fate, playing a pivotal role in diverse differentiation pathways [6,7]. Its influence is well-documented in the maturation of osteoblasts and osteoclasts to maintain bone homeostasis, as well as in neural differentiation, where it governs the transition of stem cells into structured lineages [8,9,10,11,12]. In the cardiovascular system, KLF2 is the primary mediator of atheroprotection, typically induced by healthy laminar shear stress to maintain endothelial integrity [13]. Crucially, KLF2 is a potent anti-inflammatory agent that suppresses the NF-κB signaling pathway, thereby curtailing the production of pro-thrombotic and pro-inflammatory molecules [8,14]. However, in pro-atherogenic environments characterized by disturbed flow, KLF2 expression is silenced, leading to enhanced immune cell dysfunction and a milieu ripe for lipid accumulation.

Despite the established prominence of both PCSK9 and KLF2 in cardiovascular pathology, the potential cross-talk between these two regulators during the monocyte-to-foam cell transition remains largely unexplored. To examine this relationship, we used GGTI298, a geranylgeranyltransferase-I inhibitor associated with the expression of KLF2, and GGPP, a geranylgeranyl pyrophosphate associated with the expression of KLF2 [12,15]. Treatment with GGTI298 increased KLF2 expression and reduced PCSK9 expression under ox-LDL-induced conditions. Previous studies have independently demonstrated that KLF2 regulates inflammatory activation and lipid-related responses in monocytes and macrophages [8,13,14], whereas PCSK9 contributes to macrophage inflammation and foam cell-associated processes [5,16,17]. However, it remains unclear whether changes in KLF2 expression are associated with altered PCSK9 expression during ox-LDL-induced foam cell formation. Therefore, the potential relationship between KLF2 and PCSK9 in monocytes represents an important but poorly defined knowledge gap. The present exploratory study was designed to examine this relationship using in vitro experiments in RAW264.7 cells, together with an in silico assessment of the predicted interaction between GGTI298 and PCSK9.

If KLF2 is the guardian of the cellular inflammatory state and PCSK9 is the driver of its lipid-laden transformation, a regulatory axis likely exists between them that governs the birth of the foam cell. Given the shared transcriptional pathways and functional overlap, it is plausible that KLF2 may directly or indirectly modulate PCSK9 expression to regulate lipid handling in monocytes. In the current study, we investigated this potential regulatory role using the RAW264.7 cell line and chemical modulators of KLF2, specifically GPPP as an inhibitor and GGTI298 as an activator, by evaluating PCSK9 expression at both the mRNA and protein levels during ox-LDL-induced foam cell formation. Although KLF2 and PCSK9 have each been implicated in monocyte inflammation and lipid metabolism, their relationship during ox-LDL-induced foam cell formation remains poorly defined. Our previous study showed that the presence of GGTI298 increased KLF2 expression and was associated with reduced lipid accumulation, oxidative stress, and mitochondrial dysfunction in RAW264.7 cells exposed to ox-LDL [17]. The present exploratory study examined whether chemical treatments associated with changes in KLF2 expression also alter PCSK9 expression under similar conditions. Molecular docking was additionally used to explore whether GGTI298 could adopt a predicted binding pose within PCSK9. These experiments were intended to generate a mechanistic hypothesis, rather than demonstrate direct transcriptional regulation, physical binding, PCSK9 inhibition, or therapeutic efficacy.

Accordingly, this exploratory study used in vitro experiments in RAW264.7 cells to examine the association between changes in KLF2 and PCSK9 expression under ox-LDL-induced foam cell formation conditions. In parallel, an in silico molecular docking approach was used to explore whether GGTI298 could adopt a predicted binding pose within PCSK9. These approaches were intended to generate preliminary mechanistic hypotheses, rather than establish direct binding, functional inhibition, or therapeutic efficacy.

## 2. Materials and Methods

### 2.1. Cell Culture and Foam Cell Induction

A murine monocyte cell line, RAW264.7, was obtained from ATCC and cultured in Dulbecco’s Modified Eagle Medium (DMEM) supplemented with 10% fetal bovine serum (FBS) and 1% penicillin-streptomycin at 37 °C in a humidified incubator with 5% CO_2_. To induce foam cell formation, cells were incubated with oxidized low-density lipoprotein (ox-LDL) at a concentration of 50 µg/mL for 48 h.

### 2.2. Chemical Modulation of KLF2

To modulate the level of KLF2, monocytes were pretreated with either geranylgeranyl pyrophosphate (GGPP, 10 µM) to suppress KLF2 expression, or a geranylgeranyltransferase inhibitor (GGTI298, 20 µM; Cayman Chemical Company, Ann Arbor, MI, USA), known to elevate KLF2 expression. Treatments were applied 1 h before the addition of ox-LDL and maintained throughout the incubation period. 

### 2.3. Quantitative Reverse Transcriptase Polymerase Chain Reaction

Total RNA was extracted using TRIzol reagent (Invitrogen Corporation, Carlsbad, CA, USA), and cDNA was synthesized using the iScript™ cDNA Synthesis Kit (Bio-Rad Laboratories, Hercules, CA, USA). Quantitative polymerase chain reaction (PCR) was performed using SYBR Green Master Mix (Applied Biosystems, Thermo Fisher Scientific, Waltham, MA, USA) on a StepOnePlus™ Real-Time PCR System. Relative gene expression levels of KLF2 and PCSK9 were calculated using the ΔΔCt method, keeping GAPDH as an internal control. All reactions were performed in triplicate with three independent experiments.

All qRT-PCR tests were performed in technical triplicate across three independent biological experiments. Technical triplicates were averaged to obtain one value for each independent experiment; therefore, the statistical sample size was *n* = 3 per treatment group.

### 2.4. Immunocytochemistry and Confocal Microscopy Imaging

RAW264.7 cells were cultured on coverslips placed in 6-well plates. Following treatment, the cells were fixed with 4% paraformaldehyde for 15 min, permeabilized with 0.1% Triton X-100, and blocked with 5% bovine serum albumin for 1 h at room temperature. PCSK9 was detected using a rabbit polyclonal anti-PCSK9 primary antibody at a dilution of 1:200, followed by an Alexa Fluor 488-conjugated secondary antibody at a dilution of 1:500. Nuclei were counterstained with DAPI.

Images were acquired at a resolution of 1024 × 1024 pixels, using a pinhole size of 1.0 Airy unit, a scan speed of 400 Hz, and twofold line averaging. Laser power, detector gain, offset, zoom, and all acquisition parameters were maintained consistently across treatment groups. Laser power, detector gain, offset, pinhole size, zoom, and scanning parameters were kept identical for all experimental groups. Five randomly selected, non-overlapping fields were acquired from each sample. PCSK9 fluorescence was quantified using ImageJ software, version 1.54p. For each field, the PCSK9-positive area was selected, and the integrated fluorescence intensity was measured after subtraction of the mean background fluorescence obtained from a cell-free region of the same image. The background-corrected fluorescence intensity was then normalized to the number of DAPI-positive nuclei in that field. The normalized values from five fields were averaged to obtain one value for each sample. Data were obtained from three independent experiments.

The bracketed Leica parameters should be copied directly from the LAS X image metadata (April, 2025) to avoid reporting estimated settings.

### 2.5. In Silico Analysis for Determining the Interaction Between GGTI298 and PCSK9

#### 2.5.1. Protein Selection and Preparation

The three-dimensional crystal structure of human proprotein convertase subtilisin/kexin type 9 (PCSK9) was obtained from the RCSB Protein Data Bank PDB ID: 6MV5. Among the available chains, chains A and H were identified as containing the critical ligand-binding region, specifically residues GLU1 to LYS214. These relevant chains were isolated for molecular docking studies, and extraneous elements such as water molecules, additional chains, and co-crystallized ligands were removed using Discovery Studio Visualizer 2021 [18]. The refined protein structure was then energy minimized in SWISS-PDBViewer software, version Linux 64, OS X, to eliminate steric clashes and ensure proper atomic geometries [18]. Structural validation and visualization were further performed using PyMOL software version 2.5.4 [19] to assess the integrity of folding and side-chain orientation.

#### 2.5.2. Ligand Preparation

The 2D chemical structure of GGTI298 was downloaded from the PubChem database, version 4.1, with the compound ID: 441406 [20]. This structure was then converted to PDB format using Open Babel, an open-source chemical toolbox that facilitates format interconversion [21]. Subsequently, the ligand was protonated, partial atomic charges were assigned using the Gasteiger method, also known as the Gasteiger–Marsili method, which is a commonly used empirical technique in computational chemistry for calculating partial atomic charges in molecules, and the file was converted into PDBQT format using AutoDock Tools version 1.5.6 [22]. To further enhance conformational stability before docking, energy minimization of the ligand was conducted in PyRx, version 1.2, using the MMFF94 force field [23].

#### 2.5.3. Molecular Docking Procedure

Molecular docking simulations were performed using AutoDock Vina, version 1.2 5 a widely used docking algorithm known for its speed and accuracy [24]. A grid box was centered over the known ligand-binding region of PCSK9, guided by prior mutagenesis and structural studies. Grid dimensions were defined to encompass the entire active site, and an exhaustiveness value of 8 was applied to ensure adequate conformational sampling. Multiple binding poses were generated and ranked based on the predicted binding free energy. The most energetically favorable pose (lowest ΔG) was selected for downstream interaction analysis.

#### 2.5.4. Post-Docking Interaction Analysis

The top-ranked docking pose of the GGTI298-PCSK9 complex was examined in both PyMOL, version 3.10, and Discovery Studio Visualizer, a 3D experience platform [19]. Key non-covalent interactions, such as hydrogen bonds, hydrophobic contacts, van der Waals interactions, and π–π stacking, were identified and mapped to specific residues within the active site. The binding affinity of the ligand was recorded as ΔG (in kcal/mol), providing a quantitative measure of interaction strength.

### 2.6. Statistical Analysis

Data are presented as mean ± standard error of the mean from three independent experiments. Data distribution was assessed using the Shapiro–Wilk normality test. For normally distributed data, differences among multiple groups were analyzed using one-way analysis of variance followed by Tukey’s multiple-comparisons test. If the data did not meet the normality assumption, the Kruskal–Wallis test, followed by Dunn’s multiple-comparisons test, was used. A value of *p* < 0.05 was considered statistically significant.

## 3. Results

### 3.1. Effects of GGPP and GGTI298 on KLF2 and PCSK9 Expression Under ox-LDL-Induced Conditions

Quantitative RT-PCR was performed to examine KLF2 and PCSK9 expression in RAW264.7 cells following ox-LDL exposure and chemical induction and inhibition. The addition of ox-LDL significantly reduced KLF2 expression and increased PCSK9 expression compared with the untreated control group. The addition of GGPP was associated with a further reduction in KLF2 expression and an increase in PCSK9 expression. In contrast, the addition of GGTI298 increased KLF2 expression and reduced PCSK9 expression relative to the ox-LDL-treated group.

**Figure 1:**
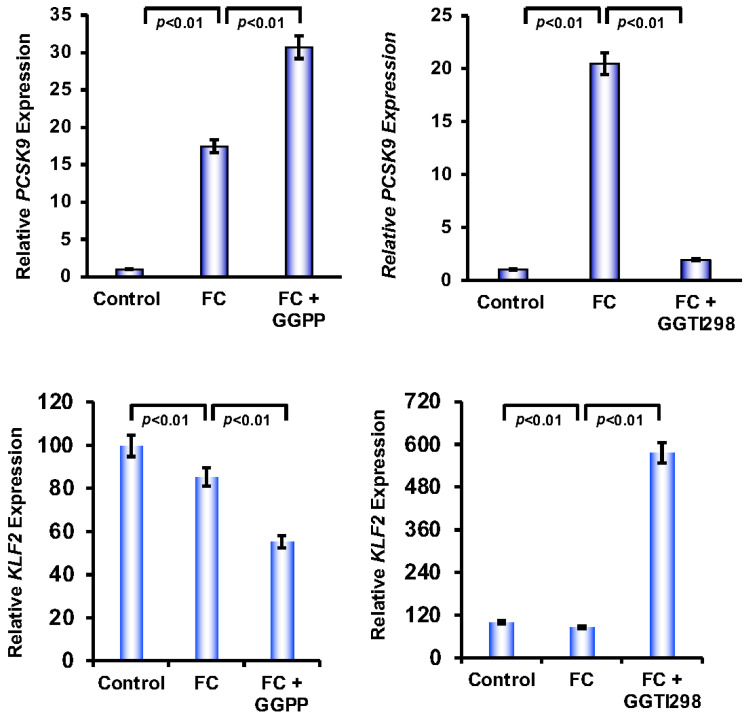
Effects of GGPP and GGTI298 treatment on KLF2 and PCSK9 expression under ox-LDL-induced conditions. Quantitative reverse-transcription polymerase chain reaction analysis showed that treatment of RAW264.7 cells with oxidized low-density lipoprotein increased PCSK9 expression and reduced KLF2 expression. Treatment with GGPP further reduced KLF2 expression and increased PCSK9 expression, whereas treatment with GGTI298 increased KLF2 expression and reduced PCSK9 expression. Data are presented as mean ± SEM from three independent experiments (*n* = 3). Statistical significance was determined using one-way ANOVA followed by Tukey’s post hoc test. Abbreviations: GGPP, geranylgeranyl pyrophosphate; GGTI298, geranylgeranyltransferase-I inhibitor 298; KLF2, Krüppel-like factor 2; PCSK9, proprotein convertase subtilisin/kexin type 9; ox-LDL, oxidized low-density lipoprotein; SEM, standard error of the mean; ANOVA, analysis of variance. Data are presented as mean ± SEM from three independent biological experiments (*n* = 3). Each qRT-PCR sample was analyzed in technical triplicate, and the technical replicates were averaged before statistical analysis.

These inverse changes indicate an association between KLF2 and PCSK9 expression under the experimental conditions used. However, because GGPP and GGTI298 affect protein geranylgeranylation and multiple downstream signaling pathways, these findings do not establish that the observed changes in PCSK9 expression are exclusively mediated by KLF2. Data are presented as mean ± SEM from three independent biological experiments, with each qRT-PCR test performed in technical triplicate. Statistical significance was determined using one-way ANOVA followed by Tukey’s post hoc test (Figure 1).

### 3.2. Effect of GGPP Treatment on PCSK9 Protein Expression Under ox-LDL-Induced Conditions

**Figure 2:**
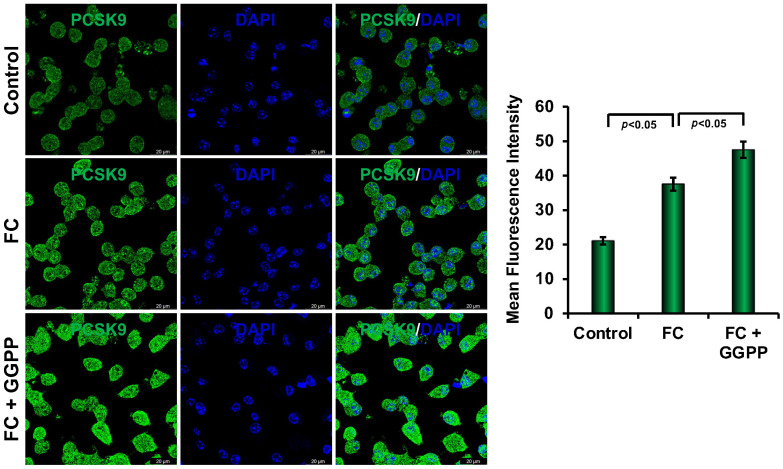
Effect of GGPP treatment on PCSK9 protein expression under ox-LDL-induced conditions. Representative confocal microscopy images showed that ox-LDL treatment increased PCSK9 protein expression in RAW264.7 cells. PCSK9 expression increased further following GGPP treatment. Nuclei were counterstained with DAPI. Quantification of mean fluorescence intensity confirmed the increase in PCSK9 protein expression. Data are presented as mean ± SEM from three independent experiments (*n* = 3), with five randomly selected fields analyzed per sample. Statistical significance was determined using one-way ANOVA followed by Tukey’s post hoc test. Abbreviations: GGPP, geranylgeranyl pyrophosphate; PCSK9, proprotein convertase subtilisin/kexin type 9; ox-LDL, oxidized low-density lipoprotein; DAPI, 4′,6-diamidino-2-phenylindole; MFI, mean fluorescence intensity; SEM, standard error of the mean; ANOVA, analysis of variance.

Immunocytochemical analysis showed that the addition of ox-LDL significantly increased PCSK9 protein expression in RAW264.7 cells compared with the untreated control group. PCSK9 fluorescence intensity increased further in cells treated with GGPP together with ox-LDL (Figure 2).

These findings show that the addition of GGPP was associated with reduced KLF2 expression and increased PCSK9 protein expression under the conditions examined. However, the results do not establish that the increase in PCSK9 was directly caused by KLF2 reduction because GGPP can affect multiple geranylgeranylation-dependent pathways. Data are presented as mean fluorescence intensity ± SEM from three independent biological experiments, with five randomly selected fields analyzed per sample. Statistical significance was determined using one-way ANOVA followed by Tukey’s post hoc test.

### 3.3. Effect of GGTI298 Treatment on PCSK9 Protein Expression Under ox-LDL-Induced Conditions

**Figure 3:**
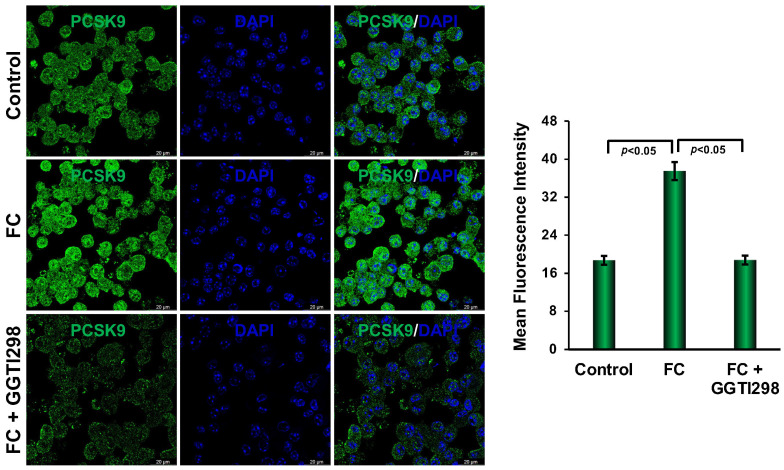
Effect of GGTI298 treatment on PCSK9 protein expression under ox-LDL-induced conditions. Representative confocal microscopy images showed that the addition of ox-LDL increased PCSK9 protein expression in RAW264.7 cells, whereas the addition of GGTI298 reduced PCSK9 expression. Nuclei were counterstained with DAPI. Quantification of mean fluorescence intensity confirmed the reduction in PCSK9 protein expression following GGTI298 treatment. Data are presented as mean ± SEM from three independent experiments (*n* = 3), with five randomly selected fields analyzed per sample. Statistical significance was determined using one-way ANOVA followed by Tukey’s post hoc test. Abbreviations: GGTI298, geranylgeranyltransferase-I inhibitor 298; PCSK9, proprotein convertase subtilisin/kexin type 9; ox-LDL, oxidized low-density lipoprotein; DAPI, 4′,6-diamidino-2-phenylindole; MFI, mean fluorescence intensity; SEM, standard error of the mean; ANOVA, analysis of variance.

Immunocytochemical analysis showed that the addition of ox-LDL significantly increased PCSK9 protein expression compared with the untreated control group. The addition of GGTI298 significantly reduced PCSK9 fluorescence intensity relative to the ox-LDL-treated group (Figure 3).

These findings demonstrate that the addition of GGTI298 was associated with increased KLF2 expression and reduced PCSK9 protein expression under the experimental conditions used. Nevertheless, the reduction in PCSK9 cannot be attributed exclusively to KLF2 because GGTI298 inhibits geranylgeranyltransferase-I and may affect additional signaling pathways. Data are presented as mean fluorescence intensity ± SEM from three independent biological experiments, with five randomly selected fields analyzed per sample. Statistical significance was determined using one-way ANOVA followed by Tukey’s post hoc test.

### 3.4. Predicted Molecular Docking Pose of GGTI298 Within PCSK9

In silico molecular docking studies revealed that GGTI298 binds favorably to the catalytic domain of PCSK9, exhibiting a binding energy of −7.5 kcal/mol. This level of affinity reflects a moderately strong and biologically relevant interaction, comparable to those reported for small-molecule inhibitors of PCSK9 in earlier studies [25,26].

**Figure 4:**
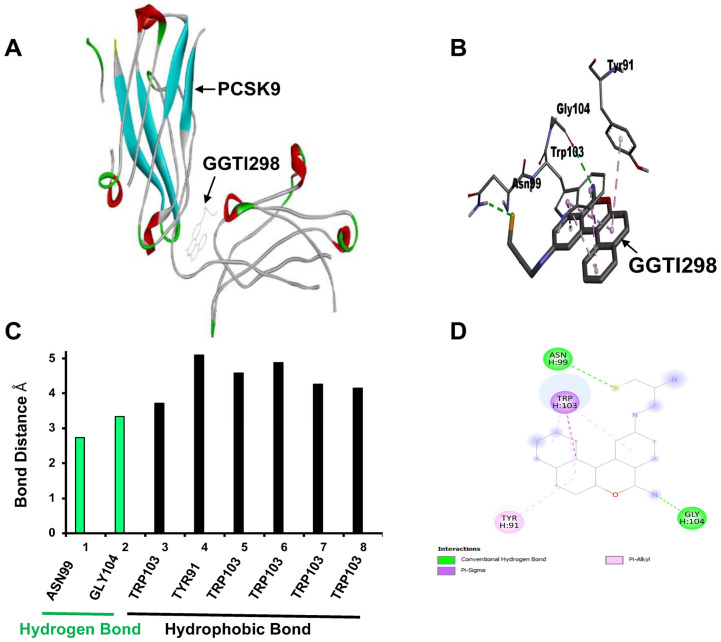
Structural insights into the interaction between GGTI298 and PCSK9 (PDB ID: 6MV5). (**A**) Surface representation of the predicted docking pose of GGTI298 within a potential binding region of PCSK9. (**B**) Three-dimensional representation of the predicted contacts between GGTI298 and surrounding amino acid residues. (**C**) Predicted interaction distances, including hydrogen bond contacts with Asn99 and Gly104 and hydrophobic or π interactions involving Tyr91 and Trp103. (**D**) Two-dimensional interaction map showing the predicted residues surrounding GGTI298. The calculated docking score was −7.5 kcal/mol. These findings represent computational predictions and do not establish direct binding, inhibition of PCSK9 activity, or disruption of the PCSK9–LDLR interaction. Abbreviations: GGTI298, geranylgeranyltransferase-I inhibitor 298; PCSK9, proprotein convertase subtilisin/kexin type 9; LDLR, low-density lipoprotein receptor; Asn, asparagine; Gly, glycine; Tyr, tyrosine; Trp, tryptophan.

Molecular docking predicted that GGTI298 could adopt a favorable pose within a potential binding pocket of the modeled PCSK9 structure, with a calculated docking score of −7.5 kcal/mol. The interaction analysis indicated predicted hydrogen bonds with Asn99 and Gly104 and hydrophobic or π interactions involving Trp103 and Tyr91 (Figure 4). 

These contacts represent computational predictions and do not confirm direct binding, PCSK9 inhibition, or disruption of the PCSK9–LDLR interaction. Direct biochemical and functional assays will be required to validate the predicted interaction. These polar interactions serve to further anchor the ligand in the catalytic site and enhance its binding specificity. The interaction profile of GGTI298 closely resembles that of known PCSK9-binding agents, such as therapeutic monoclonal antibodies alirocumab and evolocumab, which are known to target similar structural motifs [27].

Importantly, the binding interface of GGTI298 includes residues implicated in the interaction between PCSK9 and the low-density lipoprotein receptor (LDLR). This suggests that GGTI298 may competitively interfere with the PCSK9-LDLR complex formation, potentially reducing LDLR degradation. Consequently, these findings support the potential of GGTI298 as a non-covalent modulator of PCSK9 activity, with therapeutic relevance in conditions such as hypercholesterolemia and atherosclerosis [4,28]. 

### 3.5. Structural Visualization of the Predicted GGTI298-PCSK9 Docking Model

To further elucidate the molecular mechanism underlying the interaction between GGTI298 and PCSK9, we conducted detailed structural analyses using molecular docking simulations based on the crystal structure of PCSK9 (PDB ID: 6MV5). The docking results confirmed a well-fitted binding pose of GGTI298 within the PCSK9 ligand-binding cavity, supported by favorable binding energy (−7.5 kcal/mol) and spatial accommodation within the active site.

**Figure 5:**
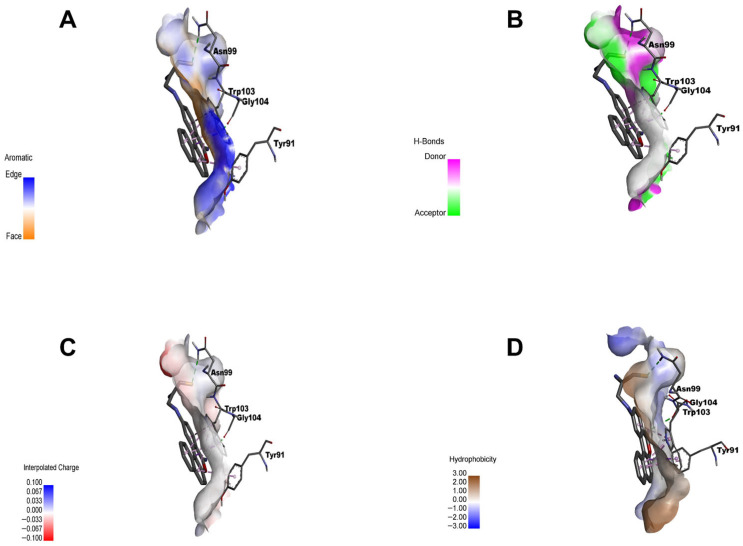
Structural interaction analysis of GGTI298 with PCSK9 (PDB ID: 6MV5). (**A**) Surface visualization of the predicted GGTI298 docking pose within a potential binding cavity of PCSK9. (**B**) Three-dimensional representation of the predicted non-covalent interactions between GGTI298 and surrounding PCSK9 residues. (**C**) Detailed representation of the predicted hydrogen bonding and hydrophobic contacts, including their calculated distances. (**D**) Two-dimensional interaction diagram identifying the amino acid residues predicted to interact with GGTI298. These molecular docking results are hypothesis-generating and require confirmation through direct biochemical and functional studies. Abbreviations: GGTI298, geranylgeranyltransferase-I inhibitor 298; PCSK9, proprotein convertase subtilisin/kexin type 9.

The aromatic surface area visualization (Figure 5A) revealed that GGTI298 is surrounded by several aromatic and hydrophobic residues within PCSK9, suggesting potential π–π stacking interactions that contribute to the conformational stabilization of the ligand–receptor complex. This aromatic landscape further supports the high-affinity nature of the binding interaction.

Figure 5B illustrates the non-covalent binding environment of GGTI298 within the PCSK9 active site. Notably, the ligand forms numerous Van der Waals contacts with residues such as Leu125, Ile129, and Phe379. These interactions collectively promote tight binding and favorable orientation of GGTI298 within the binding cleft.

A comprehensive interaction map (Figure 5C) highlights critical hydrogen bonds between GGTI298 and PCSK9 residues Lys129 and Thr377, with bonding distances ranging between 2.8 and 3.2 Å. In addition to these polar interactions, the model demonstrates the presence of other stabilizing forces, including hydrophobic contacts and electrostatic interactions, contributing to the overall affinity and specificity of binding.

Furthermore, the schematic depiction in Figure 5D identifies key residues involved in the binding interface, specifically Lys, Thr, Phe, and Ile, each annotated with the corresponding interaction type (e.g., hydrogen bonding, hydrophobic interaction, π–π stacking). This detailed residue-level analysis underscores the multi-faceted binding mechanism between GGTI298 and PCSK9, supporting its potential role as a non-covalent modulator capable of interfering with PCSK9-LDLR interactions. These visualizations are descriptive and hypothesis-generating and do not confirm direct binding, binding affinity, PCSK9 inhibition, or interference with the PCSK9-LDLR interaction.

These findings reinforce the structural plausibility of GGTI298 as a PCSK9-binding molecule, with therapeutic implications for foam cell formation of monocytes.

## 4. Discussion

The transformation of monocytes into lipid-laden foam cells is an important early event in the development of atherosclerotic lesions. While the systemic role of PCSK9 in regulating cholesterol homeostasis is well-documented, its localized impact on cellular differentiation and vascular inflammation is a burgeoning field of study. This study provides preliminary evidence of an inverse association between KLF2 and PCSK9 expression under ox-LDL-induced conditions. Our findings demonstrate that ox-LDL significantly induces PCSK9 expression in RAW264.7 cells, a process that is strikingly modulated by the status of KLF2. Specifically, our findings show that chemical treatments associated with changes in the expression of KLF2 produced an inverse change in PCSK9 expression under ox-LDL-induced conditions, suggesting that KLF2 may contribute directly or indirectly to the regulation of PCSK9.

The induction of PCSK9 by ox-LDL observed in our study aligns with previous reports suggesting that lipid loading creates a feed-forward loop of inflammation and lipid uptake. PCSK9 is known to enhance the expression of scavenger receptors such as CD36 and LOX-1, while simultaneously promoting the degradation of the LDL receptor (LDLR) and inhibiting cholesterol efflux via the ABCA1 transporter [1,2,5]. The canonical function of the enzyme PCSK9 has been the degradation of the receptor of hepatic LDL, leading to elevated levels of plasma LDL cholesterol [4,29]. It was also reported that PCSK9 exacerbates foam cell formation, not only by increasing lipid accumulation, but also by promoting an inflammatory monocyte phenotype [30]. Our results show that ox-LDL exposure significantly increased PCSK9 mRNA and protein expression; however, the present study does not establish that PCSK9 directly controls monocyte differentiation or foam cell formation. This is consistent with the findings that PCSK9 expression in monocytes is stimulated by ox-LDL through the dynamic activation of the mitochondrial ROS pathway, further linking metabolic stress to PCSK9 production [16].

An important finding of our study is the inverse association between KLF2 and PCSK9 expression. KLF2 is an established regulator of vascular homeostasis that limits NF-κB signaling and supports an anti-inflammatory cellular environment [15]. Our data show that the addition of GGPP was associated with reduced KLF2 expression and increased PCSK9 expression, suggesting an inverse relationship between these molecules under the experimental conditions used. This relationship is critical, as KLF2 is typically downregulated in regions of low shear stress, the very regions prone to plaque formation. Reduced KLF2 expression may be associated with increased PCSK9 expression; however, the present findings do not establish a direct transcriptional relationship between KLF2 and PCSK9. This regulatory mechanism mirrors the role of KLF2 in other differentiation models, such as osteoclastogenesis and neural maturation, where KLF2 serves to maintain a progenitor or quiescent state and prevent aberrant differentiation [9,31].

To further examine this chemical association, we used GGTI298, a geranylgeranyltransferase-I inhibitor that increased KLF2 expression under our experimental conditions. The reduction in PCSK9 expression following the addition of GGTI298 was associated with increased KLF2 expression, although the findings do not establish that this effect is exclusively KLF2-dependent or that it prevents a pro-atherogenic phenotype. This is particularly relevant given that current PCSK9 therapies, such as the monoclonal antibodies alirocumab and evolocumab, primarily target circulating PCSK9 to lower systemic LDL [27]. However, these systemic therapies do not necessarily address the intracellular production of PCSK9 within the vascular wall. Our findings suggest that changes in KLF2 expression may be associated with altered PCSK9 expression; however, the biological and therapeutic significance of this relationship requires further validation, such as the suppression of VCAM-1 and MCP-1 [6]. It has also been shown to influence monocyte function, inflammation, and the osteoclast differentiation process by regulating various pathways [7,8,9,15,31,32].

The docking analysis predicted that GGTI298 could adopt a possible binding pose within PCSK9, with a calculated docking score of −7.5 kcal/mol; however, this does not demonstrate direct binding or functional inhibition. The interaction model predicted hydrogen bond contacts with Asn99 and Gly104 and hydrophobic or π interactions involving Tyr91 and Trp103. These observations generate the hypothesis that altered PCSK9 expression may represent one pathway associated with the addition of GGTI298, but direct KLF2-dependent transcriptional regulation, PCSK9 binding, and effects on LDLR degradation were not demonstrated that aim to interfere with the protein’s hotspot for LDLR binding [33]. Current monoclonal antibody therapies like alirocumab and evolocumab target circulating PCSK9, but do not directly impact PCSK9 expression within vascular tissues, where it plays a role in plaque instability [34]. Our findings propose that targeting KLF2 could complement these therapies by reducing the expression of PCSK9 intracellularly, which also strengthens the earlier report of vascular cells and smooth muscle cells [17]. Genetic KLF2 knockdown, overexpression and rescue experiments, PCSK9 promoter-reporter assays, and chromatin immunoprecipitation studies will be required to establish whether the observed effects are KLF2-dependent and whether KLF2 directly regulates PCSK9 transcription. 

The molecular docking analysis indicated that GGTI298 could adopt a favorable predicted binding pose within a potential binding region of PCSK9, with a calculated binding energy of −7.5 kcal/mol. However, this computational result should not be interpreted as evidence of direct physical binding or functional inhibition. The identified hydrogen bonding and hydrophobic interactions represent predicted molecular contacts generated by the docking model. Therefore, whether GGTI298 binds PCSK9 under physiological conditions or affects PCSK9-LDLR interactions remains unknown. Direct binding assays and functional studies examining LDLR abundance, PCSK9-LDLR interaction, and LDL uptake will be required to validate this hypothesis. 

This study identifies an inverse association between KLF2 and PCSK9 expression under ox-LDL-induced conditions. Our findings show that chemical treatments associated with changes in KLF2 expression produced inverse changes in PCSK9 expression, but direct suppression of PCSK9 by KLF2 and prevention of lipid accumulation were not demonstrated in the present study. The observed changes in KLF2 and PCSK9 expression following the addition of GGTI298 provide a basis for further investigation, but therapeutic efficacy and functional PCSK9 inhibition remain to be established. Further genetic, biochemical, functional, and in vivo studies are required to determine the mechanistic and translational significance of these observations.

This study has a few limitations. First, the experiments were performed only in RAW264.7 cells, and the findings require validation in primary murine macrophages and human monocyte-derived macrophages. Second, genetic loss-of-function and overexpression/rescue experiments were not performed to establish whether the observed changes in PCSK9 are specifically dependent on KLF2. Third, direct functional measurements of foam cell formation, including lipid accumulation, ox-LDL uptake, intracellular cholesterol, cholesterol efflux, and expression of lipid-handling proteins, were not included. Finally, the molecular docking results represent computational predictions and do not confirm direct binding or inhibition of PCSK9. Further biochemical, cellular, and in vivo studies are required to validate these observations.

The study used a single immortalized murine monocyte cell line and requires validation in primary murine and human monocytes. GGPP and GGTI298 have broad effects on protein geranylgeranylation, and the observed changes in PCSK9 cannot be attributed exclusively to KLF2. KLF2 knockdown, overexpression, and rescue studies are required to establish causality. The current study did not directly measure lipid uptake, intracellular cholesterol, cholesterol efflux, or the expression of CD36, LOX-1, and ABCA1. Finally, the docking findings are computational predictions and do not confirm direct GGTI298–PCSK9 binding or functional inhibition.

## 5. Conclusions

This study uncovers the role of KLF2 as a key transcriptional suppressor of PCSK9 during foam cell formation. Our in vitro data show that ox-LDL-induced PCSK9 expression is counteracted by the activation of KLF2, underscoring the protective role of KLF2 in limiting lipid accumulation and inflammation by monocytes. Additionally, GGTI298 not only enhances the expression of KLF2, but also directly interacts with PCSK9 at its catalytic domain, suggesting a dual mechanism of action. Whereas current PCSK9 therapies focus on extracellular inhibition, KLF2-targeted strategies may offer a more integrated approach by reducing PCSK9 production within the vascular wall. This may enhance lipid clearance and dampen local inflammatory responses, ultimately limiting foam cell formation and plaque progression. Future in vivo studies on the KLF2-PCSK9 axis will determine the therapeutic potential of chemical activators like GGTI298 in managing cardiovascular disease. Such dual-action compounds represent a promising avenue for targeting both systemic lipid regulation and vascular inflammation at the cellular level.

This exploratory study demonstrates that chemical treatments associated with changes in KLF2 expression produce inverse changes in PCSK9 expression under ox-LDL-induced conditions in RAW264.7 cells. These findings suggest that PCSK9 regulation may represent one pathway associated with the previously reported effects of GGTI298 on lipid accumulation, oxidative stress, and mitochondrial dysfunction during foam cell formation [17]. However, the present study does not establish direct transcriptional regulation of PCSK9 by KLF2, direct binding or functional inhibition of PCSK9 by GGTI298, or a causal role for PCSK9 in reducing foam cell formation. Further genetic, biochemical, functional, and in vivo studies are needed to test this proposed relationship.

## Data Availability

The corresponding author will provide the necessary data supporting the findings of this study upon a reasonable request. The authors are accountable for ensuring the continued availability of the data.
